# Dentistry and the Microscope

**Published:** 1857-03

**Authors:** 


### DENTISTRY AND THE MICROSCOPE.

Much light has been thrown on anatomy, physiology and pathology by the use of the microscope, and much connected with these sciences remains yet to be investigated by means of this instrument. This is not less true of these sciences viewed in their relation to dental, than to general surgery. The attention of the dental profession is becoming more and more enlisted in microscopic observations, which, connected with a dissemination of correct chemical knowledge we regard as the most efficient means of perfecting dental science. An important step in this direction was the establishment of a chair of "Microscopic and Comparative Anatomy of the Teeth," in the Baltimore College. We, however, do not see where there is room for a course on the microscope, in a session so short, if the other departments receive the attention they require, and would hence suggest to the several schools the propriety of longer sessions.

The Introductory Lecture of Prof. Johnson is, in the main, interesting, instructive, and satisfactory. Unfortunately, he weakens its influence and his own, almost at the outset, by travelling out of his subject, outside of science, and beyond the precints of reliable history, to make an uncalled for attack on the character of one who, in life, was a great—good man. We allude to the following sentence:—

"But at length Michael Servetus, a Spaniard, armed with all the knowledge of his predecessors, proclaimed the circulation of the blood in a theological tract (Christianismi Restitutio, etc.), in 1553, for which tract, however, he was burned at the stake by Calvin, in Geneva, a few months after its publication."

We were struck with the remark of a friend on reading this passage; and we believe that all unbiassed minds will draw the same inference from it·

"The connection and the manner in which this is introduced, is prima facie evidence of the incompetency of its author to render an impartial judgment in the case. No impartial man would go so far out of his course, to drag in an item which, even if true, could throw no light upon his subject."

For the sake of the sciences which they teach, public instructors should not thus discredit themselves.

---

☞We have endeavored to be accurate in sending bills, but mistakes may have occurred. Should any one who has paid receive a bill, he will please notify us, and we will at once correct the mistake. If any one who has paid for the present volume is omitted in our published receipts, we make the same request of him.

---

### WESTERN DENTAL SOCIETY.

This young and vigorous society holds its next meeting in Saint Louis on Wednesday, the 20th of May next. We have no programme setting forth the probable order of business; but if we may take the Chicago meeting as an index of character, the society will not be idle during its session. We wish the society a full and happy meeting, and hope to be present ourselves to enjoy the feast of reason.

---

### COMMENCEMENT EXERCISES.

The commencement exercises of the Ohio College of Dental Surgery were held on Wednesday evening, Feb. 25th in the public lecture room of the college.

The exercises were opened with prayer and reading a portion of the sacred scriptures, by Rev. B. P. Ayelott, M. D., D. D., President of the board of trustees, after which he delivered an addres on "The Professional Studies of the Dental Pupil," presented a copy of the Holy Bible to each of the graduates, and conferred on them the degree of "Doctor of Dental Surgery." The valedictory address to the graduates was delivered by the Dean of the faculty, his subject being "The Dental Profession and its Appropriate Work." The audience was then dismissed with the benediction.

---

### L'ART DENTAIRE.

Revue Mensuelle de la Chirugie et de la prothese Dentaires.

Par M M. Fowler et Preterre, Dentistes Americains a Paris.

We have just received the January number, the first issue, of this new monthly. It contains thirty-two pages of original and select matter, and its articles are somewhat copiously illustrated with woodcuts, among which are represented Dr. J. D. White's dynamometer, and Putnam's instruments for producing local anasthaesia.

The proceedings of the "American Dental Convention," are commenced in this number and are to be continued. The price for the United States, is 25 francs.

---

### DENTAL COLLEGES.—CLASSES OF 1856—'57.

By the politeness of Prof. P. H· Austen, we are able to lay before our readers the names of the graduates and under graduates of the "Baltimore College of Dental Surgery;" and to state that their commencment exercises were held on Friday evening, Feb. 27th, under the most pleasant circumstances.

Graduates.—Jose Arcadio Ansley, Cuba; William Hicks Bracey, Virginia; Alex. Mills Campbell, M. D. Virginia; Joseph Dinwiddie, Jr., Virginia; Israel Francis Disosway, Virginia; James Benjamin Dubose, Alabama; Richard Davis Fleming, N. Carolina; Theodore Turner Fogle, Ga.; Conrad Gunther, Maryland; John William Hendel, Pennsylvania; Henry Hobart Keech, Maryland; Jas. Gaston Kirkpatrick, Arkansas; Henry Bliss Noble, District of Columbia; James Horace Parker, Virginia; Geo. Washington Perry, New York; John Alex. Robertson, Scotland; Daniel McCollum Rogers, South Carolina; William Green Turner, Rhode Island; John Vallerchamp, Pennsylvania; Fredrick Volck, Maryland.

Undergraduates.—John Nepomuceno Boza, Cuba; Carver Willis Brown, Virginia; Henry Clarke, Maryland; John Emory Day, Pennsylvania; Edward Franklin Evans, Georgia; James Orlando Fitch, Connecticut; Dixon Sasnett Hall, Alabama; Edward Daniel Hamner, Virginia; Jacob Zollinger Hoffer, Pennsylvania; James Madison Hopson, Kentucky; William

Randal Johnston, M. D., Tennessee; Alvis Haynes Jones, North Carolina; Robertus Kennon Lea, Alabama; Nimrod Washington Long; Alabama; William Seaton Magruder, Virginia; Sidney Gorham Marshall, N. York; George Woodson Monroe, N. Carolina; Jules Lacoste Pochon, Maryland; Charles Frederick Sheafer, Pennsylvania; William Eugene Smith, South Carolina; Thornton Wm. Tomlinson, Virginia; Vines Edmund Turner, North Carolina; Alexander Preston Vaughan, Kentucky; Milton Clay Vaughan, Kentucky; George Porter Woodbury, Massachusetts.

Graduates 20; Under graduates 25; Class of session 1856—'57, 45.

Below we give the Class of the "Ohio College of Dental Surgery," and hope, before going to press, to be able also to report that of the "Pennsylvania College."

Graduates.—Char'es J. May, Illinois; G. W. Hall, Kentucky; Henry A. Smith, Ohio; Charles Silsbee, Ohio; Albert G. Philips, Ohio.

Juniors.—M. W. Wartman, Canada West; D. L. Overholser, Indiana; William J. Herrit, Ohio; Wiliiam R. Lilly, Ohio; John N. Emery, Pennsylvania; Cephas C. Dills, Ohio; S. L. Bracey, Mississippi; Nixon Hildreth, Ohio; N. W. Williams, Ohio; Geo. W. Barrere, Ohio; John M. Moorehead, Ohio; P. R. Slingsby, Wisconsin; N. P. McCafferty, Wisconsin; William T. Allison, Indiana; J. L. Springgate, Illinois; Charles W. Robinson, Ohio; James L. Kemper, Ohio; William A. Ross, Indiana.

Graduates 5; Juniors 18; Class of 1856—'57, 23.

---

### THANKS.

We take this opportunity to return our thanks to the Mississippi Valley Association for the liberalty of its members in defraying the expenses of reporting for the Register, the discussion at its late meeting. We thought that neither we nor the profession could afford to do without the report, and, therefore, made arrangements to obtain it, as we supposed, at our own expense; but the association decreed otherwise, and we are most thankfully resigned to the dissappointment.

---

### BROACHES.

We have received from J. D. Chevalier of N. Y., some fine broaches, of various sizes; both smooth and barbed, for removing nerves from the fangs of teeth, preparing the canals etc.

They are set in small neat ivory handles—this is quite an addition; it enables the operator to handle them with more dexterity and precision. Every dentist who fills the fangs of teeth should have a complete assortment of these, as it is impossible to perform this class of operations without the appropriate instruments.

☞ Just as our last form was going to press, we receivhd the proceedings of the "St. Louis Dental Society," we were, therefore, unable to place them under their appropriate head. This brief account of the society shows that our brethren in St. Louis, are not only alive, but wide awake. We have not yet received the *proceedings* of the "Cincinnati Dental Society." In fact, the society has not *proceeded.*

---

The articles in the original department of the present number do not appear exactly in the order which we desired; but, to delay the press as little as possible, we concluded, in the language of *expectant* medicine, to "let nature have its course;" and, hence, they appear in rotation, about as we received them.

---

*The Dental Obturator.*

The obturator for December is back to its old size again and is not in the least exhausted by its late gigantic and successful effort. The article of Dr. Knapp, on "Sensibility of Dentine" is interesting and practical. We have not room, or we would lay it before our readers. The editor's articles on "Change of locality" and "What does it all amount to" contain many interesting thoughts. His "Chips" are less interesting, and we believe he had better leave off *chopping.* The Obturator is practical, pointed and spicy; but occasionally over does the thing, and becomes somewhat *flat.* For example, it falls down and tramps on itself when, under the head of "Wants," it tries to make sport of somebody's "capillary attraction" notions, while at the same time it sets forth "*cohesive* attraction" as an agency concerned in the retention of plates in the mouth. Nothing could well be more ridiculous than the latter idea; but the writer has simply forgotten the meaning of cohesion. We hope to be able in a future number to give some space to selections from the Obturator.

---

*The North American Medico-Chirurgical Review.* Edited by S. D. Gross, M. D., Prof. of Surgery in the Jefferson Medical College, and T. G. Richardson, M. D., Prof. of Anatomy in the Medical Department of Pennsylvania College, Philadelphia.

The Review is a bi-monthly journal, of a hundred and sixty pages. It results from a union of the interests of the "Philadelphia Medical Examiner" and the "Louisville Review." This journal makes a fine appearance, and evidences alike the taste of its publishers, the industry and energy of its editors, and the ability of its contributors. It is published by J. B. Lippincott & Co., No. 20 North Fourth street, Philadelphia, at $4 00 per annum, in advance, or $5 00 at the end of the year.

---

*Comparative Anatomy of the Teeth.*

Our readers will be gratified to learn that a work with the above title is being prepared by Prof. C. B. Chapman, M. D., of the Ohio College of Dental Surgery. The work is desired and needed by the Dental Surgeon, the physician, the naturalist, and every lover of science; and it is a source of gratification to all that the right man has undertaken the task of preparing it. We hope the Doctor may have the health and leisure necessary to its speedy completion.

